# Urban Green Space and Subjective Well-Being of Older People: A Systematic Literature Review

**DOI:** 10.3390/ijerph192114227

**Published:** 2022-10-31

**Authors:** Tianrong Xu, Nikmatul Adha Nordin, Ainoriza Mohd Aini

**Affiliations:** Centre for Sustainable Planning and Real Estate (SUPRE), Faculty of Built Environment, Universiti Malaya, Kuala Lumpur 50603, Malaysia

**Keywords:** urban green space, subjective well-being, older people, green space characteristics

## Abstract

A growing number of articles have identified and reported the benefits and importance of urban green spaces for improving human well-being, but there is a significant knowledge gap regarding the impact of urban green spaces on the subjective well-being of older adults. The literature search (August 2015–August 2022) was derived from two major scientific databases, Google Scholar, and Web of Science. As a result, 2558 articles were found, 1527 of which were retrieved from WOS and the rest from Google Scholar. Bibliometric methods and VOSviewer software were used to screen and organize the articles in the relevant fields. Finally, 65 articles met the review criteria. The included studies aim to capture the benefits of various features of urban green spaces in meeting or enhancing the subjective well-being needs of older adults. The results of our review further support the existence of a strong link between older adults’ subjective well-being and various features of urban green spaces, providing new insights for future in-depth reexamination and policy development. Furthermore, the relationship between urban green spaces and older adults’ subjective well-being depends not only on the urban green spaces themselves but also on the characteristics of the older adult population that uses them.

## 1. Introduction

Urbanization and aging are occurring and growing at an unprecedented rate and are key challenges for most countries worldwide. Urbanization leads to more people living in urban areas [1], and urban overcrowding can exacerbate the risk of infectious diseases [2]. Globally, there has been a significant demographic shift, with 16.2% of the global population projected to be aged 65 years and over by 2050 (United Nations, New York, NY, USA, 2020). Older people are defined by the World Health Organization as those over 60 years of age, with those 60 to 74 years of age being younger seniors, 75 to 89 years of age being seniors, and 90 years of age or older being long-lived seniors [3]. The United Nations have identified the health and well-being of older people as one of the most urgent social issues of the present day. Aging research represents a new frontier in terms of making the most of older people’s resources while highlighting their needs and potential contributions. Evidence from research has demonstrated that healthy, active, and positive aging can be achieved through lifestyle changes and effective interventions [4]. Focusing on the concept of well-being is fundamental to happiness and aging [5] and in the common interest of human development [6].

Research on the origins of subjective well-being was first prevalent in the fields of educational psychology, gerontology, marital success, and epidemiology. Diener (2012) coined the term subjective well-being as people’s evaluation of their own lives, well-being, and a sense of purpose [7]. Subjective well-being is not a single phenomenon, and scientists believe it needs to be studied in terms of both the affective component (positive and negative emotions) and the cognitive component (life satisfaction). This has some relevance to the quality of life as defined by the World Health Organization Quality of Life Group [3]. The social indicators movement of the 1960s and 1970s saw a gradual increase in interest in the study of subjective well-being by experts and scholars in various fields, and subsequently, the terms goodness of life, satisfaction, happiness, and quality of life were used interchangeably [8]. Research also suggests that different domains of well-being may significantly contribute to favorable life outcomes [9], that people with more positive affect report better social relationships and healthier behaviors [10], and that people with high subjective well-being have stronger immune systems, live longer and have lower mortality from cardiovascular disease [11], have fewer sleep problems, are more capable of self-regulation and coping [12], and are relatively more cooperative and pro-social [13]. Maintaining high levels of subjective well-being is a fundamental aspect of successful aging. Subjective well-being does not decline with age, and empirical studies have shown that older people do not have lower levels of subjective well-being compared to younger people. Subjective well-being can be assessed in gerontology by measuring self-esteem, life satisfaction, and happiness.

Urban green spaces have been identified as being relevant to promoting human health and enhancing well-being [5]. In recent years there has been increased interest in research concerning the health-promoting potential of urban parks [14]. Green corridors not only raise the species richness of landscape patches but also help sustain daily physical activity and positively impact human health [15]. Urban green spaces provide spaces to experience nature [16], with potential associations with health through three pathways: hazard reduction, encouragement of physical activity [17], and resiliency [18]. Recommendations from a meta-analysis have shown that environmental factors such as the quality and accessibility of green spaces can have an impact on the physical activity of citizens [19]. Many countries are keen to highlight the value of urban green space utilization. In Denmark, for example, approximately 25% of health policies state that urban green space has a positive impact on the health and well-being of the population [20]. Furthermore, studies have revealed that facilities such as lighting, sidewalks, benches, or plant species richness in urban green spaces also influence the use and activity of urban green spaces [21], with a positive relationship with the mental health of users [22]. Interestingly, some studies have used social media data in combination with traditional field surveys and questionnaires for future research, an approach that could provide researchers with a wealth of detail about older visitors’ personal experiences, behaviors, and their approach to visiting parks [23].

A framework for the potential impact of urban green space on mental health has been succinctly summarized, from attention recovery theory to the sense of place framework to stress reduction theory. Evidence from an epidemiological perspective also implicates urban green spaces in improving residents’ general physical and mental health [24]. Some researchers also acknowledged that increased social interactions within urban green spaces are associated with capacity building, especially among vulnerable groups (e.g., older people), and green spaces in cities can be associated with improved overall well-being [25] and self-perceived health status [26], suppressed morbidity and increased life expectancy [27], and increased satisfaction with life prospects [28], among other ways to promote healthy aging in older people [29]. The perception of spatial places is multidimensional and results from a large number of meaningful human interactions with physical entities or physical spaces, with the perceived value being influenced by time and environment [30]. The health and well-being of older people are influenced not only by their behaviors but also by the environment in and around which they live. Parks are important places for outdoor recreation, socialization, and physical activity and are significant environmental resources that support the well-being of older people and healthy aging, with studies showing that 10% of park visitors are older people [31] and that their visits to parks can mitigate stress and reduce anxiety and depression [18]. A study of older people in the UK found that older people preferred parks with a variety of public facilities but without nuisance [11]. In addition, negative aspects of urban green spaces are now an obvious concern for researchers in the literature, such as health-related ecosystem damage [32], air quality issues [33], and safety issues [34].

Despite reasons to believe in the positive impact of urban green spaces on human health (including mental health) and well-being, and the significance of their use, due to the comprehensive relationship between urban greenspace use and subjective well-being, these articles did not conclusively demonstrate which characteristics of urban green space play a constructive role in improving the subjective well-being of older people, which is a particularly significant omission. Therefore, the main goal of this systematic review is to fill this need: seeking to understand the association between specific characteristics of green space and the subjective well-being of older people to help urban designers improve the design of urban green spaces and to help governments develop green space policies that are responsive to vulnerable groups. In pursuit of our primary objective, we also investigated a second objective, namely the influence of socio-demographic characteristics of different older people regarding their perceived green space and self-reported well-being. We expect these findings to develop further a deeper understanding of the relationship between urban green spaces and the health and well-being of older adults.

To this end, this paper aims to address the following research questions:

1. Which characteristics of urban green spaces may influence older people’s subjective well-being?
2. Which sociodemographic characteristics have implications for older people’s access to subjective well-being and perceptions of urban green spaces?

## 2. Methodology

### 2.1. Search Methods

In this study, a systematic search of the literature review was conducted. A systematic review is a research method that aggregates multiple studies based on specific criteria to provide high-quality evidence [35]. We selected articles from August 2015 to August 2022 from Google Scholar and Web of Science databases for the review. To capture the entire knowledge landscape, we further completed this search using the most common synonyms found in the field of study. The captured results were then combined to identify our final research subjects. This study formalizes the importance of three characteristics of urban green spaces in influencing subjective well-being in old age. The final search syntax was: TITLE-ABS-KEY = (“green space”) OR (“green area”) OR (“public space”) OR (“parks”) OR (“open space”) AND TITLE-ABS-KEY = (“the elderly”) OR (“older people”) OR (“older person”) OR (“aging”) OR (“Senior citizens”) AND TITLE-ABS-KEY = (“well-being”) OR (“human well-being”) OR (“subjective well-being”) OR (“aging”) OR (“Senior citizens”). 

### 2.2. Inclusion and Exclusion Criteria

In August 2015, the Second World Assembly on Aging was held by the World Health Organization in Madrid, Spain, and the Global Report on Aging and Health was released, formally introducing the critical concept of healthy aging. Therefore, it is significant that the starting point for inclusion in the review was defined in August 2015, as mentioned earlier. The review aims to examine the important theoretical contributions since 2015 on the role of urban green spaces in influencing the subjective well-being of older people and in establishing insights for future research. Our review study follows the topical information of the times, fits the key trends of social development, and provides a scientific basis for promoting the sustainable development of aging and the aging industry. Clarifying the research cycle of the paper was the first inclusion criterion of this review study.

The inclusion criteria were (1) English-language articles published from August 2015 to August 2022; (2) peer-reviewed studies; (3) surveys or qualitative studies; and (4) the primary research focused on urban green spaces and the subjective well-being of older adults. The exclusion criteria were (1) articles published before August 2015 and after August 2022; (2) editorials, opinion pieces, and non-research papers; (3) non-English language articles; and (4) research content not related to urban green spaces or the well-being of older adults, such as the well-being of young people.

### 2.3. Search Outcomes

**Figure 1:**
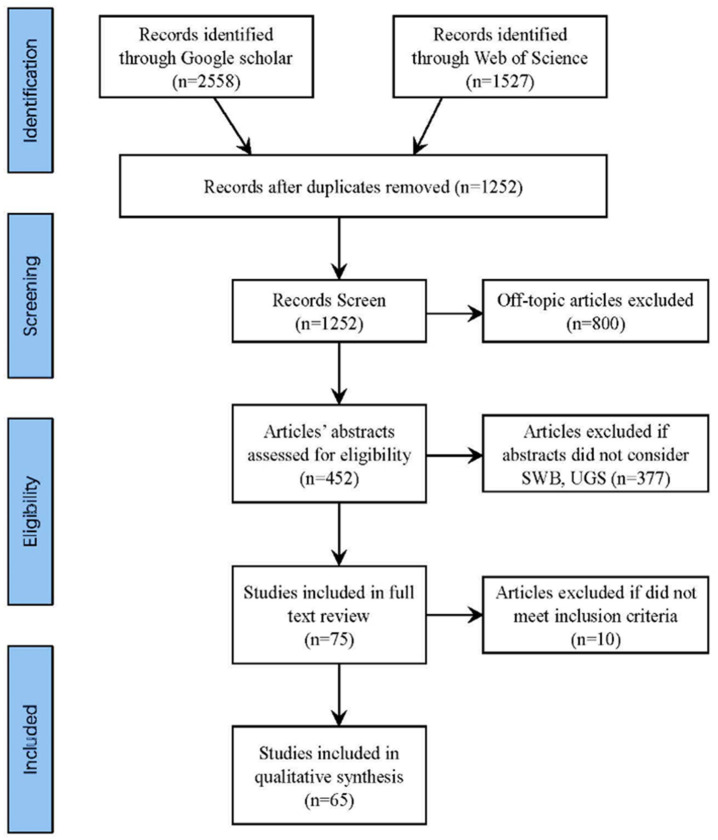
The flow diagram of the study.

Of the total number of publications surveyed, 2558 references were from Google Scholar and 1527 references from Web of Science. Approximately two-thirds of the literature was removed because it depended heavily on the relationship between the variables (e.g., insufficient information). After excluding these studies, 452 articles were left to be read in detail. Next, conceptual studies or studies without a relevant empirical component were excluded to obtain more evidence with empirical support. Finally, 65 articles were included in the literature review analysis for thematic analysis. Most of these articles were from English language journals, with five being in Chinese. More information about these studies is detailed in Figure 1.

### 2.4. Data Extraction

Based on the search outcomes, three authors extracted the data and resolved disagreements by consensus. Extracted data included sample characteristics (i.e., region, country, size), study design (i.e., qualitative study, quantitative study, mixed study), spatial characteristics of urban green spaces, green characteristics of urban green spaces, green features of urban green spaces, and socio-demographic characteristics of the elderly. Table 1 illustrates detailed information on the various characteristics of urban green spaces and the sociodemographic characteristics of older people.

### 2.5. VOSviewer and Visual Plot

VOSviewer is a free JAVA-based software focusing on visualizing scientific knowledge [36]. The most significant advantages of VOSviewer over other bibliometric software are its powerful graphical visualization capabilities [37], its application to large-scale data [38], and its versatility in adapting to various databases [39] and various formats of source data [40].

VOSviewer software was used to analyze and assess the hotspots and knowledge clusters of this study. By importing the bibliometric dataset, visual plots of keywords co-occurrence analysis, common country network analysis, and coupling analysis can be generated.

**Figure 2:**
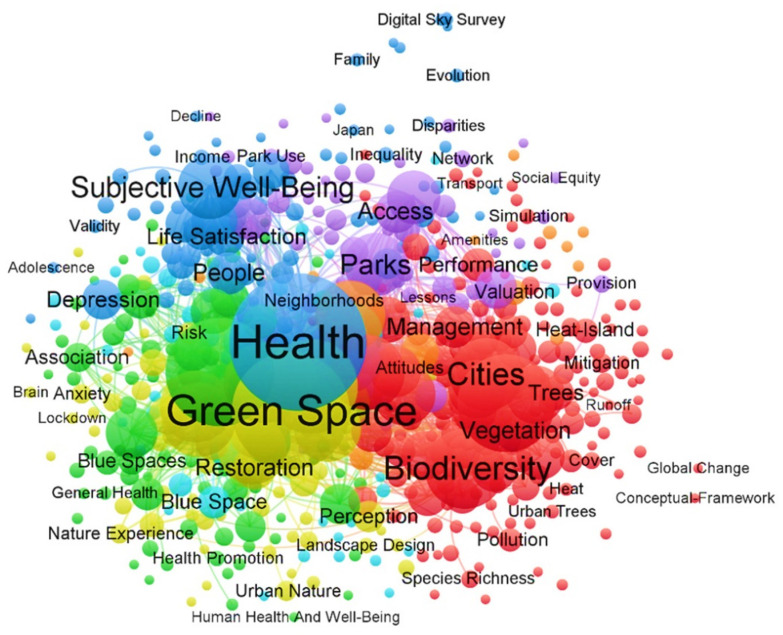
Keywords co-occurrence analysis 1.

**Figure 3:**
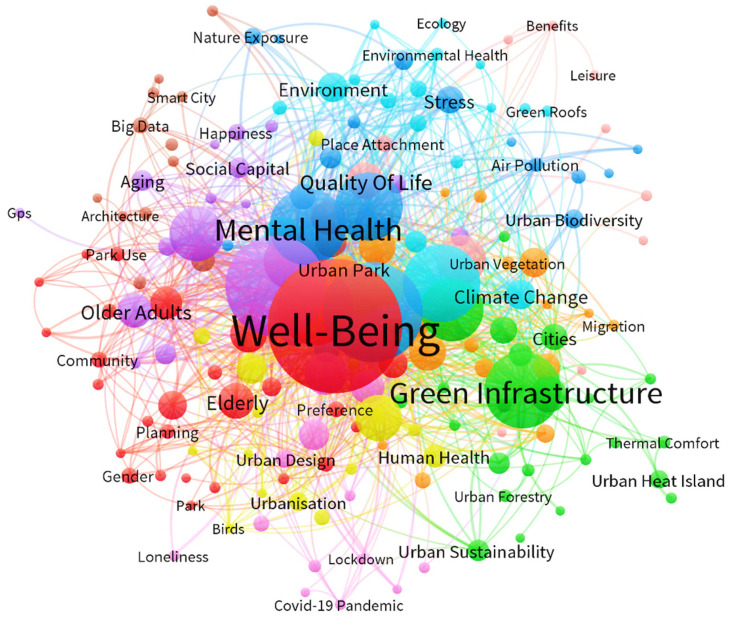
Keywords co-occurrence analysis 2.

The visualization of the term maps is provided in both Figure 2 and Figure 3. Figure 2 demonstrates the main terms related to green space. Figure 3 illustrates the main terms related to well-being in the selected articles. Each term is represented by a circle, the size of which reflects the number of publications in which the term was found, while the distance between every two terms provides key indicative information about the relevance of the term. The color indicates the group of terms with relatively strong relevance. In these two visualizations, it is evident that a high degree of symbiotic relationship exists between well-being, health, subjective well-being, green space, green infrastructure, and biodiversity. Clustering the strength of correlation between these terms provides us with the evolutionary path of disciplinary knowledge and the research hotspots and trends in this field for the next review studies, which helps to further identify the research objects and observation topics. 

**Figure 4:**
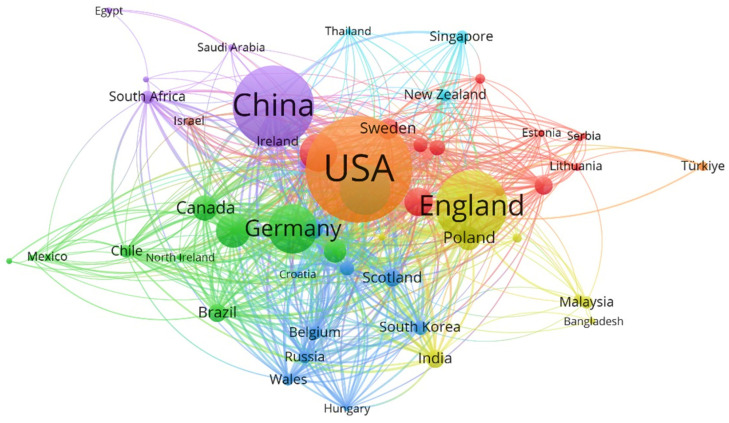
Analysis of co-countries networks (Source: authors).

Figure 4 shows the mapping analysis of the network of cooperating countries. It reflects the cooperation between countries in the research on the related areas of urban green space and the well-being of the elderly, as well as the degree of cooperation. The larger the node, the more productive the research is in this area. The length and thickness of the links between nodes represent the collaborative relationships between countries. The country with the highest total link strength is the United States, followed by China and the United Kingdom.

**Figure 5:**
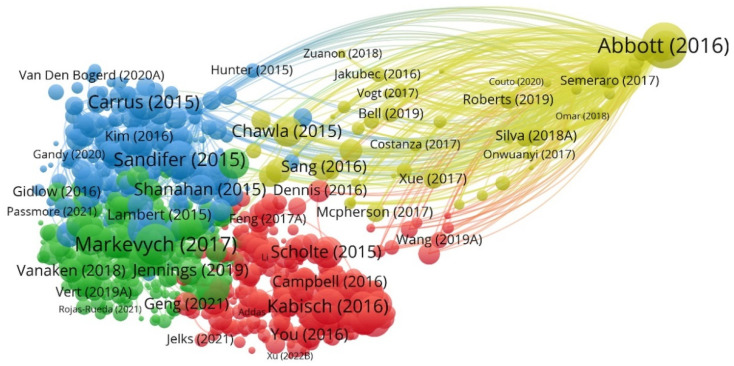
Bibliographic coupling analysis (Source: authors) [17,41,42,43,44,45,46].

Figure 5 displays the graphical analysis of bibliographic coupling. Experts from sociology, psychology, gerontology, epidemiology, urban design, and land-landscape planning attach great importance to the intersection of green space and well-being. Moreover, what is visible in this visualization is that most scholars are more connected to each other and have very strong collaborative relationships. Only a small number of scholars have published independently.

This study not only presents the associations and results between three main characteristics of urban green space and the subjective well-being of older people but also analyzes the relationship between the perception of green space and the subjective well-being of older people from the perspective of sociodemographic characteristics. There is no doubt that only a few articles met the review criteria, mainly because many studies were conducted on younger people and were inconsistent with measures of well-being. 

## 3. Findings

The results of the study provided a comprehensive analysis of the literature from two major databases (Google Scholar and Web of Science) since August 2015 that are highly relevant to the research topic. The results of the review are presented in two themes to reflect the influence of three main characteristics of urban green spaces (spatial, green, and gray characteristics) and sociodemographic characteristics on the perception of green spaces and the subjective well-being of older people.

This section first presents the findings on the study characteristics of the two research themes. The second section describes the findings of the study on the correlation between spatial characteristics, green characteristics, and gray characteristics of urban green spaces and the subjective well-being of older people.

### 3.1. Study Characteristics

The first research theme was to investigate the association between the spatial characteristics (Table 2), green characteristics (Table 3), and gray characteristics (Table 4) of urban green spaces and the subjective well-being of older adults, respectively. A total of 50 articles were included in the review. Of these, 48% of the articles (*N* = 24) originated from European countries; with 22% (*N* = 11) and 20% (*N* = 10) being from Asian and American countries, respectively; the remaining 10% of the articles (*N* = 5) belonged to Australia. Close to half of the studies relate to Europe. However, the locations of South America and Africa were excluded from these studies. From a research content perspective, four articles concerned the three main characteristics of urban green spaces [47,48,49,50]. There were two articles each on the spatial characteristics and gray characteristics of urban green spaces [51,52] and green characteristics and gray characteristics of urban green spaces [53,54], and only one article was published on the spatial characteristics and green characteristics of urban green spaces [55]. The rest of the articles were analyzed only in terms of one characteristic of urban green spaces, spatial characteristics (*N* = 19), green characteristics (*N* = 13), and gray characteristics (*N* = 9). Seen from the perspective of research methods, quantitative research methods, including surveys, questionnaires, and regression analysis, were used in 68% of the papers (*N* = 34). Qualitative research methods, including bibliometric methods, case studies, interviews, and meta-analysis, were applied in 30% of the papers. A mixed approach of social prescribing approach was conducted in only 2% of the papers (*N* = 1). In addition, the study sample sizes varied from 15 to 36,368 individuals. All study characteristics for the first research theme are shown in Supplementary File S1.

The second research theme was to examine the influence of sociodemographic characteristics of older people on their perception of green space and subjective well-being. A total of 15 articles were included in the review. Five articles (30%) originated from European countries (Switzerland, the Netherlands, Poland, and Germany); the remaining 10 articles were from China (*N* = 3), the United States (*N* = 3), and Australia (*N* = 4). Moreover, our review revealed that the survey method was the most conducted research method (53.33%). In addition to the survey method, reviews, case studies, cohort studies, ordinal regression, and bibliometric methods were also widely applied in this area of research. Study sample sizes ranged from 100 to 24,954 individuals. All study characteristics for the second research theme are shown in Supplementary File S2.

### 3.2. Urban Green Space Characteristics and Subjective Well-Being of Older People

Table 2 illustrates the total of 26 articles [2,6,47,48,49,50,51,52,55,56,57,58,59,60,61,62,63,64,65,66,67,68,69,70,71,72] is included in the review on the study of the correlation between spatial characteristics of urban green spaces and the subjective well-being of older adults. Among them, 16 articles belong to research articles (RA) forming the highest percentage (61.54%), while the rest are articles (23.08%, *N* = 6), systematic reviews (11.53%, *N* = 3), or original scholarship (3.85%, *N* = 1). Area, size, type, location, distance to park, quantity, quality, availability, accessibility, safety, frequency, and duration as spatial characteristics of urban green spaces are used to decipher their association with the subjective well-being of older people. It is worth noting that accessibility is considered the most significant green space feature affecting the subjective well-being of older adults, as it is mentioned and analyzed in detail in 17 articles (65.38%). Accessibility is identified as essential place support to encourage older people to participate in green space and engage in physical activity. 

Secondly, more than half of the papers report that the type (50%, *N* = 13) and size (53.8%, *N* = 14) of parks are remarkably connected to the experience and perception of green space among older adults. The richness of green space type and scale is described in detail as providing diverse spaces for physical activity and social interaction and increasing the likelihood that older adults would use green space. Over 40% of the articles emphasize the many benefits of park size (42.3%, *N* = 11), distance to home (46.2%, *N* = 12), and quantity (46.2%, *N* = 12) on older adults’ mental health. The larger the area of green space and the closer the distance to home, the more significant the health benefits of green space. An increase in the number of parks shows a positive correlation with psychological well-being. In addition, more than 30% of the articles reveal correlations between the frequency of greenspace visits and duration of stay (38.5%, *N* = 10), quality (34.6%, *N* = 9), location (30.8%, *N* = 8), and availability (30.8%, *N* = 8), and the impact of these characteristics on older adults’ self-reported health and well-being. 

Park location and availability positively correlate with the frequency of greenspace visits and duration of stay among older people. Providing and maintaining good urban green space quality is an effective strategy to moderate the health benefits and enhance the well-being of the elderly. Finally, only four articles (15.3%) mention the perception of safety. However, the final review shows that most articles combine the dimension of the perceived safety of green spaces and gray features of green spaces, perhaps due to the interesting finding that older adults use more gray facilities (e.g., paths and accessibility) in green spaces when visiting them. The above results highlight that the spatial characteristics of urban green spaces can provide benefits and specific uses for older adults and can influence potential benefits for older people, such as higher levels of social support.

**Table 2:** Spatial characteristics of urban green space and the subjective well-being of older people.

The First Author (Year)	Country	Design	Participants	Urban Green Space Characteristics (Spatial Characteristics)											Subjective Well-Being of Older People	Nature of Study
				Area	Size	Type	Location	Distance	Quantity	Quality	Availability	Accessibility	Safety	Frequency and Duration	Association	
Bertram (2015) [58]	Germany	Web survey	485 usable observations		√		√	√			√	√			○	RA
Cao (2015) [72]	USA	Campbell’s model and survey	1303 responses		√						√	√			○	RA
Ettema (2016) [51]	The Netherlands	Online survey	258 questionnaires were returned									√			○	RA
Akpinar (2016) [59]	Turkey	Questionnaire	420 participants	√	√			√		√				√		RA
Rioux (2016) [60]	France	Interviews	90 adult participants	√	√	√			√					√	○	RA
Larson (2016) [61]	USA	Telephone survey	160,000 independent telephone surveys	√		√			√	√		√			○	RA
Sara Tilley (2017) [62]	UK	Experimental design	43 participants aged 65 years and over		√	√	√	√			√	√		√	○	A
White (2017) [71]	UK	Omnibus survey	7272 persons (16% aged ≥ 65years)			√									○	RA
Levy-Storms (2018) [52]	USA	Bibliometric approach	48 articles	√	√	√	√	√		√		√	√	√	○	SR
Macintyre (2019) [57]	UK	Cross-sectional	15 older adults	√	√	√	√					√			○	A
Ayala-Azcárraga (2019) [47]	Mexico	Social prescribing program	Vulnerable populations (Includes older people)	√	√	√		√	√	√		√	√	√	○	RA
Zhang (2019) [63]	Singapore	Household questionnaire survey	1000 adults	√	√	√		√	√			√		√	○	A
Miralles-Guasch (2019) [48]	Spain	Multilevel regression analysis	269 participants (aged ≥ 60 years)						√	√	√	√			○	A
Nishigaki (2020) [64]	Japan	Multilevel cross-sectional	126,878 older adults (aged ≥ 65 years)						√						○	A
Dennis (2020) [65]	UK	Regression analyses	61.93% (aged ≥ 60years)	√	√	√			√	√		√			○	RA
Shuvo (2021) [66]	Australia	Poisson regressions	Sydney (2776) Singapore (2630) Dhaka (5071)						√			√	√		○	OS
Nguyen (2021) [67]	Australia	Bibliometric approach	68 articles from 59 studies	√	√	√	√	√	√	√					○	SR
Palliwoda (2021) [49]	Germany	Questionnaire	more than 1700 users		√		√				√				○	RA
Farfán Gutiérrez (2021) [56]	Mexico	On-screen visual interpretation method	828,079 people living in the study area	√				√	√		√	√			○	A
Roberts (2021) [68]	UK	Linear mixed effects models	10,000 residents			√	√	√				√			○	RA
Sharifi (2021) [6]	Australia	Descriptive statistics	36,368 residents		√			√	√	√	√	√			○	RA
Gianfredi (2021) [2]	Italy	Bibliometric approach	34 articles	√		√		√			√	√		√	○	SR
Petrunoff (2021) [69]	Singapore	Interviewer-assisted survey	3435 Participants		√					√				√	○	RA
Veitch (2022) [50]	Australia/Belgium	Cross-sectional	501 older adults (aged ≥ 65 years)											√	○	RA
Ali (2022) [55]	India	Field survey	500 persons (38.4% aged ≥ 60 years)				√	√	√	√		√	√	√	○	RA
Oviedo (2022) [70]	Canada	Survey and questionnaire	140 persons (16% aged ≥ 60 years)			√									○	RA

Note RA: Research Article; SR: Systematic Review; A: Article; OS: Original Scholarship. √ denotes the relevant variables appearing in the paper; ○ Indicates that the relevant variables appearing in the paper are associated with the subjective well-being of older people.

A total of 20 articles [1,47,48,49,50,53,54,55,63,73,74,75,76,77,78,79,80,81,82,83] were included in the review of the study on the correlation between green characteristics of urban green spaces and subjective well-being of older adults, reporting 11 analyses related to green characteristics of urban green spaces. For a description of the included articles and analyses, see Table 3. Although the nature of the studies is more diverse in this area, the highest proportion of research articles remains (35%, *N* = 7). The literature review in this area reveals that 90% of the articles (*N* = 18) identify biodiversity conservation as a green feature of urban green spaces and as the green indicator most strongly associated with the subjective well-being of older adults. There is a strong correlation between perceived biodiversity and measures of the health of older adults. This is followed by vegetation richness, an indicator mentioned in 65% of the articles (*N* = 13), where rich plant communities not only support and promote biodiversity in green spaces but also promote positive socio-ecological outcomes in green spaces. Purifying the environment and providing aesthetic spaces are considered essential features of urban green spaces that can significantly contribute to ecosystem services and promote the physical and mental health of the elderly, a message we observed in 45% (*N* = 9) of the papers. In addition, improved urban microclimate (35%, *N* = 7), water resources (35%, *N* = 7), bird and animal species richness (35%, *N* = 7), and socio-cultural integration with landscape ecology (35%, *N* = 7) are also widely noted green features of green spaces as effective factors that can enhance well-being at the sensory levels of sight, hearing, and touch, reduce anxiety levels, and can promote more positive responses. Maintaining carbon and oxygen balance is assessed and analyzed by 30% of the papers (*N* = 6) as the primary green feature of urban green space, and it is concluded that maintaining carbon and oxygen balance reveals the correlation between urban green space and subjective well-being of older adults from an ecological perspective. Moreover, 25% of the papers (*N* = 5) mention urban noise as one of the main threats affecting the self-reported well-being of older adults and urban green space as a natural buffer zone. Its noise reduction function can directly or indirectly affect the life satisfaction and subjective well-being of older people. Finally, it is fortuitous that a logistic regression study from China (396 valid questionnaires returned) examines earthquake mitigation as a green indicator, which is the only article (5%) that mentions an association between earthquake mitigation and older adults’ well-being. The results of the review suggest that green features of green spaces have a non-negligible role in reducing environmental pollution, reducing life stress, relieving pain, improving sleep, providing visual stimulation, and improving mental health. The findings of this review on studies related to the green characteristics of urban green spaces and the well-being of older people provide a theoretical basis for future research.

**Table 3:** Green characteristics of urban green space and the subjective well-being of older people.

The First Author (Year)	Country	Design	Participants	Urban Green Space Characteristics (Green Characteristics)											Subjective Well-Being of Older People	Nature of Study
				Maintaining Carbon and Oxygen Balance	Purifying the Environment	Improving the Urban Microclimate	Reducing Urban Noise	Disaster Prevention and Mitigation	Biodiversity Conservation	Vegetation Richness	Water Resources	Birds and Animal Species Richness	Providing Aesthetic Spaces	Social, Cultural, and Ecological Interactions	Association	
Kabisch (2015) [53]	Germany	Bibliometric approach	219 publications	√	√	√			√	√	√	√	√	√	○	R
Reid (2017) [73]	USA	RDD and cell phones and an online survey	1281 participants	√		√			√	√					○	A
Duan (2018) [74]	China	Logistic regression	396 valid questionnaires	√	√	√		√							○	RA
Aerts (2018) [1]	Belgium	Bibliometric approach	19 studies						√						○	IR
Wen (2018) [54]	Germany	PRISMA method	44 articles		√				√	√	√	√	√	√	○	SR
Zhang (2019) [63]	Singapore	Household questionnaire survey	1000 participants						√	√					○	A
Benton (2018) [75]	UK	Natural experimental study	Older people in Greater Manchester				√		√	√			√	√	○	SP
Miralles-Guasch (2019) [48]	Spain	Multilevel regression analysis	269 participants (aged ≥ 60 years)						√	√	√				○	A
Ayala-Azcárraga (2019) [47]	Mexico	Social prescribing program	Vulnerable Populations (Includes older people)		√				√	√		√			○	RP
Cameron (2020) [76]	UK	Questionnaire	414 participants						√	√	√	√	√		○	R
Sundevall (2020) [77]	Sweden	Semi-structured walking interviews	18 park users		√				√	√	√	√			○	A
Houlden (2021) [78]	UK	Bibliometric analysis	10 articles						√						○	SR
Palliwoda (2021) [49]	Germany	Observation and surveys	more than 1700 users	√	√	√	√		√				√	√	○	R
Kühn (2021) [83]	Germany	Hierarchical regression analyses	207 older adults				√		√	√					○	RA
Jabbar (2021) [79]	Malaysia	Bibliometric approach	46 studies	√	√	√	√		√					√	○	SR
Veitch (2022) [50]	Australia	Cross-sectional	501 older adults (aged ≥ 65 years)		√	√	√		√	√	√	√	√		○	RA
Ali (2022) [55]	India	Questionnaire	500 green space users	√	√	√			√				√	√	○	RA
Ha (2022) [80]	USA	Cross-sectional	6405 residents (1378 participants aged ≥ 65 years)						√	√	√	√	√		○	RA
Cottagiri (2022) [81]	Canada	Cross-sectional	26,811 urban participants (aged from 45 to 86 years)						√	√					○	RA
Wen (2022) [82]	China	Case study	elderly people (over 65) in the city was 18.8%										√	√	○	RA

Note: R: Review; RA: Research article; SR: Systematic Review; RP: Research Paper; A: Article. IR: Invited Review; √ denotes the relevant variables appearing in the paper; ○ Indicates that the relevant variables appearing in the paper are associated with the subjective well-being of older people.

Table 4 illustrates the 17 articles [47,48,49,50,51,52,53,54,84,85,86,87,88,88,89,90] on the study of the correlation between the gray characteristics of urban green spaces and the subjective well-being of older people. A total of 12 correlational analyses of gray characteristics were considered in the current review. Of these, 76.5% of the papers identify high-quality rest facilities (*N* = 13) as influential gray facility that influences older adults’ visitation and use of urban green spaces, playing an important role in attracting visitors and promoting visitation rates. In addition, we observe that pavement conditions in urban green spaces are as important as rest facilities in influencing the well-being of older adults (76.5%, *N* = 13). Path and pavement conditions are the main aspects that reflect the inclusive design of urban green spaces and park accessibility. This result suggests that paths as gray facilities in urban green spaces are strongly associated with the perceived safety and well-being of older people in green spaces. Furthermore, 64.7% of the papers (*N* = 11) assess the importance of providing accessible green spaces, as this factor determines the safety and comfort of older people engaging in physical activities and social interactions in urban green spaces. In contrast, lighting facilities (*N* = 9), safety facilities (*N* = 9), recreational facilities (*N* = 10), sanitation facilities (*N* = 10), and campus management centers (*N* = 10) are analyzed in closer proportions (52.9%, 58.8%) in terms of their relevance in supporting the health and well-being of older adults. In addition, sports facilities (*N* = 6) and directional facilities (*N* = 6) are considered necessary aspects to keep urban green spaces in good condition. Finally, only 23.5% of the papers (*N* = 4) consider that landscape facilities affect the perception of green spaces and the well-being of the elderly.

### 3.3. Socio-Demographic Characteristics of Older People and Subjective Well-Being in Urban Green Space

Table 5 shows the 15 articles [67,91,92,93,94,95,96,97,98,99,100,101,102,103,104] included in the reviewed studies on the topic of sociodemographic characteristics of older adults and their perceived well-being in green spaces. From a demographic perspective, the differential impact of the perception of green space on health and well-being is an important element of previous studies. The second research theme focused on the sociodemographic characteristics of older adults and the influence of personal factors and family variables on their health and well-being.

**Table 4:** Gray characteristics of urban green space and the subjective well-being of older people.

The First Author (Year)	Country	Design	Participants	Urban Green Space Characteristics (Gray Characteristics)												Subjective Well-Being of Older People	Nature of Study
				Car Parking Facility	Resting Facility	Recreational Facility	Sports Facility	Sanitary Facility	Lighting Facility	Security Facility	Directional Facility	Landscape Facility	Management Buildings	Accessible Facility	Pavement Condition	Association	
Kabisch (2015) [53]	Germany	Bibliometric approach	219 publications	√	√			√	√		√		√	√	√	○	R
Ettema (2016) [51]	The Netherlands	Online survey	258 questionnaires were returned	√		√	√	√		√			√	√	√	○	RA
Levy-Storms (2017) [52]	Belgium	Bibliometric approach	48 articles		√	√			√	√	√		√	√	√	○	SR
Zhai (2017) [84]	China	Observations and interviews	7319 older adults (aged ≥ 60 years)		√				√						√	○	RA
Cerin (2017) [90]	Australia	quantitative studies	42 articles		√	√		√					√		√	○	SR/MA
Cinderby (2018) [85]	UK	Case study interviews	117 (age ≥ 55 years)	√	√	√		√		√			√	√	√	○	RA
Wen (2018)	Germany	PRISMA method	44 articles	√	√	√		√		√		√	√	√		○	SR
Miralles-Guasch (2019) [48]	Spain	Multilevel regression analysis	269 participants (aged ≥ 60 years)		√	√		√						√	√	○	A
Ayala-Azcárraga (2019) [47]	Mexico	Social prescribing program	Vulnerable Populations (Includes older people)		√	√	√	√	√						√	○	RP
Palliwoda (2021) [49]	Germany	Questionnaire	more than 1700 users		√		√									○	RA
Li (2020) [99]	China	Fuzzy Delphi questionnaire and empirical cases	30 valid questionnaires were collected		√	√	√	√	√	√		√	√	√		○	RA
Ottoni (2021) [86]	Canada	Observational and semi-structured interviews	43 older adults (2017, n = 27; 2019, n = 16)		√				√		√				√	○	RA
Kou (2021) [87]	China/UK	Interviews	20 older adults (aged 60 years and over)	√	√	√	√	√		√	√		√	√	√	○	RA
Polko (2022) [105]	Poland	Survey questionnaires	394 park users						√	√	√	√	√	√	√	○	RA
Veitch (2022) [50]	Australia/Belgium	Cross-sectional	501 older adults (aged ≥ 65 years)			√	√			√	√		√	√	√	○	RA
Rahm (2022) [89]	Sweden	Questionnaire	106 participants (Aged 18–84 years)						√							○	OA
Kimic (2022) [88]	Poland	Questionnaire	394 park users (including 69 older people)		√			√	√	√		√		√	√	○	A

Note: R: Review; RA: Research article; SR: Systematic review; MA: Meta-Analysis; A: Article; RP: Research Paper; OA: Original Article. √ de-notes the relevant variables appearing in the paper; ○ Indicates that the relevant variables appearing in the paper are associated with the subjective well-being of older people.

Similar to the content validation studies on measures of well-being in older adults, it was emphasized that age, gender, marital status, education, pre-retirement employment, and income need to be considered as essential factors in evaluating well-being in older adults. Cohort studies in the United States and Australia have indicated that psychological distress and general health deficits are more prevalent among older adults, women, and those with low education, low income, or not working. In a cross-sectional study in the Netherlands, which included age, marital status, education, and income as background variables for older people, these demographic characteristics were found to be associated with high scores in all quality-of-life domains, while women scored lower in physical health and psychological aspects. Studies in China have also suggested that older people who continue to work after retirement have better economic and physical health than those who do not work after retirement, but their mental health is lower than the latter. In terms of gender-related perceptions, men and women expressed different expectations and sensitivities regarding their perceptions and experiences of urban green spaces and public spaces, with women being more willing to spend time in the community and to be more integrated into the community’s green space.

### 3.4. In Summary: Urban Green Spaces Support Subjective Well-Being in Older People

Using thematic analysis, we grouped the collected articles according to topics: (1) Quantifying the characteristics of urban green spaces, including their spatial, green, and grey characteristics. We analyzed the positive and negative effects of the different characteristics of urban green spaces on the subjective well-being of older people. (2) Reviewed the sociodemographic characteristics of older people and their effects on subjective well-being. Based on our review, we can conclude that urban green spaces would be a corollary to the positive association in promoting health-related behaviors and subsequent positive health outcomes among older adults. Changes in sociodemographic characteristics may also contribute to the fact that older adults have inconsistent perceptions, preferences, and use of urban green spaces. Although the review methodology and exclusion conditions differ, our results are consistent with recent findings. Urban green spaces are an important factor affecting the physical and mental health of older adults [2] and are a central component of urban sustainability and resilience [106]. In the context of the growing urban aging population in the world, our review study further emphasizes the importance of integrating urban green spaces into urban planning and public health policies.

## 4. Discussion

This paper presents the findings of a review investigating the relationship between urban green spaces and subjective well-being from the perspective of older people. Despite a growing body of articles identifying and reporting on the benefits and importance of urban green spaces in improving human well-being, the available evidence on the role of urban green spaces in influencing older people’s subjective well-being is under-captured, leading to potential gaps in our understanding of older people’s use and perception of urban green spaces concerning their physical health, psychological well-being, and subjective well-being. Which characteristics of urban green spaces may influence older people’s subjective well-being, and what is the impact of sociodemographic characteristics on older people’s perceived urban green spaces on their subjective well-being? 

### 4.1. The Relationship between Different Urban Green Space Characteristics and the Subjective Well-Being of Older People

#### 4.1.1. Spatial Characteristics of Urban Green Spaces

The preconditions that influence the use of urban green spaces are their size, quantity, and quality [106]. In general, the quantity of nearby public green spaces is associated with better mental health [51,61,107], which requires urban designers to take into account the interrelationship between the community, the amount of green space, and the elderly population when conducting urban master planning. Quality is defined by describing various characteristics of green spaces, and landscape architects can demonstrate the attractiveness of green space by designing the spatial characteristics of urban green spaces [52,90,108] and is measured by natural, cultural and historical, quiet, and amenity aspects [64,66,99]. Natural spaces, especially green spaces, have clear potential and cohesive effects on reducing depression and improving mental health. Data capture through the Normalized Difference Vegetation Index or overall green cover, public policy often supports increasing the amount of green space in urban areas [65,109], as access to these green areas can improve mental health [67,110]. However, the accessibility, safety, and reachability of urban green spaces should also draw government departments and urban planners.

The choice and use of urban green spaces by older people can promote a variety of health benefits, such as increased physical health through participation in physical activities in parks in green spaces [6,68,111] and reduced tension and increased personal resilience and social cohesion through social interaction in green spaces [16]. Social cohesion is an important aspect of well-being in old age, and urban green spaces provide places and spaces for older people to enhance social cohesion and reduce the risk of mortality [2,69,112].

In addition, for older people living in more socio-economically disadvantaged communities, their consideration of the accessibility, reachability, and safety of urban green spaces also needs to be addressed. This is because the access to urban green spaces that can be identified by GIS is not necessarily applicable to older people in the analyzed areas. Examples include high-end private gardens or golf clubs. Policymakers and urban planners are keen to commission the expert design of urban green spaces. However, from the perspective of designing complexity for the real world, urban green spaces are hardly ever designed to provide any specific health benefits due to a lack of clarity about which specific features of urban green spaces provide benefits for specific groups of people, such as older people. As Lee et al. [55,70] point out in their study, there is a need to identify what health outcomes are sought and what activities in urban green spaces contribute to these outcomes. In turn, the features of urban green spaces that encourage such activities are highlighted [71,111]. Therefore, understanding and quantifying the functional and dependency relationships between urban green spaces is a fruitful strategy for promoting health effects and subjective well-being in older people.

#### 4.1.2. Green Characteristics of Urban Green Spaces

The 11 studies related to the green features of urban green spaces and the subjective well-being of older adults have been summarized in our review. Vegetation is the only primary producer in urban ecosystems and is considered to be an important component of terrestrial ecosystems and urban landscapes [113]. The natural value of public green spaces is to provide space for plant and animal habitats while supporting sustainable urban development and enhancing the health of urban dwellers by purifying space, improving microclimate, and increasing stormwater retention. Green space is not only a valuable resource for community health-related activities [1,19], but it is also an important example of resilient buffering that can reduce the incidence of cardiovascular disease and diabetes [77,114]. 

A cost-benefit and utility analysis of urban green elements from an economic perspective allow for methods and tools to economically assess the value of urban green to address all uses and co-benefits [49,115]. Urban green spaces (such as parks) are distinguished from other spaces by their unique natural systems, where people want to engage with nature and enjoy a greater variety and abundance of green elements as much as possible [78,83,116]. Long-term contact and interaction with nature parks can satisfy human needs for natural recreation and spiritual entertainment [79,117], leading to increased resilience and well-being, not only at the level of respect, acceptance, and belonging but also in terms of emotional and identity needs [55,118]. At the same time, urban ecosystems, such as green and blue spaces, also provide aesthetic services to citizens [21,80,82] and contribute to the quality of urban life [76,119].

Contact with nature, such as lushly vegetated parks or streetscapes, can promote rational mechanisms for many health benefits [74,120], which are important for preventing or improving obesity, diabetes, certain cancers, osteoporosis, and other diseases in the elderly. A spatial analysis investigating the impact of green infrastructure on health outcomes found that people aged 60+ were the only age group living in low-income areas to derive health benefits from proximity [54,65]. This indicates that the green characteristics of urban green spaces play crucial importance in the subjective well-being of older people.

To sum up, the findings of the review on the positive impact of green characteristics of urban green spaces on the subjective well-being of the elderly show that there is a strong correlation between the two, which has important design implications for urban designers and landscape planners. Designing rich plant communities is an essential design element to support the biodiversity of green spaces and a reasonable mechanism to purify the air, improve urban microclimate, and promote the health of the elderly. Increasing green natural landscapes and rich natural systems meet the requirements of the elderly for natural recreation and spiritual entertainment. Green elements can help seniors relieve stress, reduce loneliness and depression, and thus improve overall well-being. Moreover, designers need to pay attention to the implications of the richness of bird and animal species on seniors’ experience and perception of green spaces, which was an unexpected finding in our review study that urban green spaces not only provide habitats for plants and animals, but also enhance ecosystems and improve the living environment, and thus contribute to seniors’ mental health and well-being. Lastly, the aesthetic function of green spaces cannot be ignored, and designing urban green spaces that meet the aesthetic desires of the elderly is an initiative to enhance the quality of life and health, and well-being of the elderly.

#### 4.1.3. Gray Characteristics of Urban Green Spaces

The gray infrastructure of urban green spaces refers mainly to the network of facilities such as roads, accessible design, lighting systems, and street furniture in green spaces. Increased mobility has a direct impact on the quality of life of older people [85], hence the need for increased public transport use and accessible design. Research has shown that active aging schemes need to be accessed through age-friendly infrastructure, for example, with available parking or walking paths to venues [53,121]. The lack of basic facilities such as benches and paths will discourage older people from exercising in parks [84,88], especially for older people over 60 with mobility problems or disabilities. If ramps are designed without handrails, this is likely to raise safety concerns and discourage older people from continuing to visit green spaces [90,122]. The ramps are designed to be safe for the elderly and to prevent them from continuing to visit the green space [50,105]. Continued social participation requires creative approaches to promote the well-being of older and vulnerable people.

The design of urban public and green spaces, especially regarding the specific needs of older people, should be addressed [54,85] to improve the quality of life of this age group [47,48,123]. Engaging older people actively in urban life and green space and taking into account their perspectives on urban sustainability challenges is an important element promoted by inclusive design. What affects the willingness of older people (especially those with reduced mobility) to travel may be, on the one hand, transport expenditure as a burden and, on the other hand, the availability of physical infrastructure design, such as pavements, seating arrangements in public areas and ramps for the disabled. [49,87].

In addition, traffic-related air and noise pollution can directly affect the body, cause respiratory and cardiovascular health problems, and increase the risk of breast and prostate cancer. In one study, older people expressed a desire to make their experience in parks fun by ‘playing’ with exercise equipment and using other recreational facilities for stretching or even using handrails, branches, and benches to help them exercise [86,88,124]. Age-friendly facilities in parks are often designed from a functional perspective, but to support different health outcomes, urban green spaces need to be planned with a focus on activity spaces for older people and open-ended ‘play’ equipment that can be used by older people to engage in a variety of potential forms of physical activity.

Therefore, in the design of urban green spaces, attention needs to be paid to the specific needs of older people and to improve the gray infrastructure of urban green spaces through creative and friendly design and practice (e.g., a mix of interesting paths and node networks connecting open spaces can increase social cohesion and well-being). These would support the secular mobility and social interaction of older people in public and green spaces and promote healthy aging, quality of life as well as their well-being in later life.

### 4.2. Influence of Sociodemographic Characteristics on Older People’s Perception of Urban Green Spaces for Subjective Well-Being

Research has found that access to green space views, visits to urban parks and public spaces, access to private green spaces, neighborhood greening, and increased duration and frequency of green space exposure were associated with reduced stress and distress during the pandemic [125]. Studies have also found a relationship between perceived health and well-being, with the physical environment, social environment, participation opportunities, and choice of transport having a significant positive impact on the well-being of older people [121].

Over the last few decades, the quality of life of older people has been at the forefront of international policy debates on aging [126]. Health is the most relevant factor in subjective well-being, followed by income [127]. Subjective well-being is not only related to the degree of social integration, support networks, and professional activities but also social connections with parents and friends [43]. Older people with a partner [128], those with higher education and higher income [129], and those with better healthcare [130] have relatively higher subjective well-being.

Positive self-concept about health is associated with older people’s willingness to engage in physical activity, which in turn facilitates their functional autonomy. Older people who perceive positive health are more likely to enter urban green spaces for social activities and physical activity, reducing loneliness [131] and improving emotional well-being to promote subjective well-being [132]. Older people travel shorter distances, have fewer destinations to reach, and travel less by individualized means (e.g., car and bicycle), all of which are attributed to a higher incidence of mobility-limiting conditions [133]. Social isolation has become a challenge for urban older people, and urban green spaces provide open spaces for leisure and physical and social activities that benefit older people’s mental health, suggesting that social and tranquility dimensions are significantly associated with perceived resilience [134].

Some studies have shown that, across family types, multigenerational families have the lowest social capital satisfaction and self-esteem scores and the highest depression scores. This finding may be explained by intergenerational differences in political orientation or economic power leading to conflict among family members [115]. Furthermore, older people with poor health and low income tended to score higher on tests of depressive symptoms, suggesting that physical health and economic factors are associated with depression in older people. In contrast, the higher the green coverage of built-up urban areas, the lower the depressive symptoms among older residents, suggesting that a higher degree of urban greenery, a larger area of green space, better quality, and more safety can reduce the production of depressive symptoms among older residents [104]. Therefore, an increase in the area and quantity of urban green space would be encouraged in an increasingly aging society. The relationship between mental health outcomes, the proportion of green space, and socio-demographic status was demonstrated in a Tehran urban green space study that confirmed the importance of identifying older residents vulnerable to mental health problems based on the direct relationship between socio-demographic characteristics and the proportion of green space and mental health [135].

Urban green spaces positively impact residents’ physical and mental health. However, this impact may differ between older men and women due to differences in perceptions of urban green spaces between genders. The differences are in terms of safety, accessibility, species diversity, and patterns of use. Males and females also show differences in the perception and valuation of urban green space features due to the different sensitivities and expectations of males and females in terms of green space perception and experience. There are also significant differences in the perceived health and well-being of older people regarding the characteristics of urban green spaces [93].

Nevertheless, many urban sustainable greening development strategies and green justice dimensions recognize different stakeholder situations. These studies do not consider the gender equality of older people in urban green spaces. When confronting the complexity of sustainable urban greening, it is imperative to pay attention to gender equality, especially in the context of an age-friendly society, in relation to the health and well-being of older people’s perceptions of urban green spaces. Additionally, it has been observed that boosting education and eliminating inequality in residential status are more beneficial than possessing material wealth when it comes to enhancing the subjective well-being dimension [119]. Therefore, promoting re-employment, re-education, and improving the living environment of older people is more likely to promote an active perception of green space, active physical activity, and social cohesion, which is more important in promoting healthy living and well-being.

Following the literature review, we also found several studies stating that older people who choose to continue working even after retirement because of the possibility of gaining social participation and social support contribute to their meaning and purpose in life. This would help to improve their mental health and well-being and reduce their mortality rates [101].

### 4.3. Comparison of the Review Results with Previous Studies

#### 4.3.1. Applicability of Research Methodology

None of the 65 articles included in the review have been analyzed for knowledge topics with the help of visualization software. In our review process, an attempt was made to combine bibliometric methods and VOSviewer software for literature screening and organization, and visual network plots could be generated to analyze the relevance of previous studies. VOSviewer software has an absolute advantage in terms of its graphical presentation capabilities, allowing the processing of large volumes of literature in databases and enabling the construction of maps for any suitable mapping technique. On the one hand, it helps to perform scientific metric analysis and interpretation of mapping language for relevant literature data in the field of review research. On the other hand, research hotspots and relevant knowledge clusters in the cross-cutting area of urban green space and elderly well-being research can be identified. Compared with previous studies, this is a bold innovation in the research methodology aspect of this review.

#### 4.3.2. Comparison with Previous Studies

The results of our review suggest that the positive role of urban green spaces in influencing the subjective well-being of older adults can be clearly understood by establishing links between the three main characteristics of urban green spaces and the subjective well-being of older adults. Previous studies on urban green space and well-being have had different emphases and have all contributed significantly to the study of landscape well-being. However, previous studies have not categorized the characteristics of urban green spaces. Our review study, however, differs from previous studies precisely in clarifying the characteristics of urban green spaces, forming three major categories of urban green spaces: spatial characteristics, green characteristics, and gray characteristics. In addition, the discussion section provide in-depth analysis and discussion. It not only fills the gap in this theoretical knowledge but also provides references and support for subsequent studies. This is the most outstanding theoretical contribution of this paper.

In addition, most previous studies have focused on the effects of urban green space on urban citizens, and although the study sample also includes the elderly, it is not common to find articles that single out the elderly as a research group. Instead, our study focused on the effects of different sociodemographic characteristics of older adults on perceived green space and assessed well-being. Greenspace satisfaction and subjective well-being scores vary from person to person, and sociodemographic characteristics are one of the key factors in determining the impact. Thus, our review also provides theoretical support for subsequent quantitative studies.

## 5. Conclusions

Our work has further supported the understanding of the relationship between the subjective well-being of older people and urban green spaces. New insights are presented for integrating urban green spaces, subjective well-being, and older people. Specifically, two aspects are included:

(1) The spatial, green, and gray characteristics of urban green spaces not only provide space and opportunities for social interaction, alleviate anxiety and stress, and provide improvements in older people’s mood and concentration but also have a more significant contribution to older people’s subjective well-being and high levels of social cohesion.
(2) Sociodemographic characteristics also have substantial implications for older people’s subjective well-being in relation to urban green spaces. Older people’s transient perceptions, experiences, and well-being of urban green space environments are vital to the planning and design of age-friendly spaces by increasing the quantity and scale of green spaces and encouraging the optimal design of their accessibility to improve their attractiveness and suitability for older people.

## 6. Limitations and Future Research

It should be noted that there are certain limitations to the process and results of this study. Firstly, the time frame and language of article screening was the first limiting factor we encountered. We targeted our search to articles written in English between August 2015 to August 2022, which resulted in only a small number of articles (65) meeting the eligibility screening criteria. However, beyond this period, many more valuable articles on this area have been published or exist in the literature in other languages. Secondly, we conducted a literature search in a limited number of databases, such as Google Scholar and Web of Science, to present the research in a narrative synthesis. 

In addition, the inherent geographical and health diversity of older people, as well as the complex impact of socio-cultural, socio-economic background, and sociodemographic variables on the study, prevented us from understanding their different needs and differences in subjective well-being acquisition in greater detail. Furthermore, the measurement of urban green space characteristics was based on the presence of open and accessible green spaces and did not consider other factors that may have a bearing on older people’s access to health and well-being benefits, such as climatic conditions and air quality. Finally, this study did not consider the impact of private gardens or rooftop gardens at the front and back of the home on older people’s physical and mental health benefits and subjective well-being.

In future studies, we need to establish the benefits and various limiting factors such as socio-economic factors, personal values, or cultural perceptions that older people gain from visiting green spaces from the perspective of green space use patterns and individual level. In addition, an attempt could be made to ascertain if there is an effect of different types of green space features on older people’s usage preferences through the results of an eye-movement experiment. In-depth interviews could also be used to obtain completely new data on older people’s understanding of the emotional, perceptual, and experientially defined boundaries associated with enhancing urban green spaces. Finally, relevant government departments and urban planners should pay attention to and encourage policy research related to the planning and management of green space spaces for the elderly. Attention should be paid to the results of older people’s participation, experience, and perception of various features of urban green spaces and to the development of green space policies that meet the actual needs of older people and thus contribute to their well-being.

## Figures and Tables

**Table 1:** Urban green space characteristics and sociodemographic characteristics of older people.

Urban Green Space Characteristics			Sociodemographic Characteristics of Older People
Spatial Characteristics	Green Characteristics	Gray Characteristics	
Size Area Type Distance Quantity Quality Availability Accessibility Safety Frequency and duration	Maintaining carbon and oxygen balance Purifying the environment Improving the urban microclimate Reducing urban noise Disaster prevention and mitigation Biodiversity conservation Vegetation richness Water resources Bird and animal species richness Providing aesthetic spaces Social, cultural, and ecological interactions	Car parking facilities Resting facilities Recreational facilities Sports facilities Sanitary facilities Lighting facilities Security facilities Directional facilities Landscape facilities Management centers Service facilities buildings	Gender Age Household registration Marital status Religious beliefs Education level Job before retirement Income

**Table 5:** Sociodemographic characteristics and the subjective well-being of older people.

The First Author (Year)	Country	Design	Participants	Social Demographic Characteristics								Subjective Well-Being of Older People	Nature of Study
				Gender	Age	Household Registration	Marital Status	Religious Beliefs	Education Level	Job before Retirement	Income	Association	
Wang (2015) [91]	Australia	Questionnaire	319 responses	√	√		√		√		√	○	RA
Forbes (2015) [92]	Australia	National Survey	2149 adults (Aged 60–79)	√	√		√			√	√	○	RA
Ode Sang (2016) [93]	Sweden	Postal survey	1347 adults	√	√							○	RA
Gobbens (2018) [94]	The Netherlands	Cross-sectional	1031 (aged ≥ 65)	√	√		√		√		√	○	RA
Batz (2018) [96]	USA	Review	1434 articles	√	√							○	LR and MA
Janus (2019) [95]	Poland	Questionnaire	100 women aged over 60 years	√	√		√			√		○	RA
Astell-Burt (2019) [98]	Australia	Cohort study (Baseline and follow-up)	24,954 (aged ≥ 55)	√	√	√	√		√		√	○	OI
Li (2020) [99]	China	Questionnaire	9325	√	√	√			√	√		○	RA
Enssle (2020) [100]	Germany	Case study	506 questionnaires	√	√	√	√					○	RA
Massey (2021) [97]	USA	Ordinal regression analysis	1274 (aged ≥ 65 and above)	√	√					√	√	○	RA
Xie (2021) [101]	China	Large-scale survey	29,478	√	√	√	√		√	√	√	○	RA
Nguyen (2021) [67]	Australia	Bibliometric approach	68 articles from 59 studies	√	√		√		√		√	○	SR
Hackert (2021) [102]	The Netherlands	Online survey	269 (aged ≥ 65)	√	√		√	√	√	√	√	○	RA
Han (2021) [103]	USA	User survey	769 (aged ≥ 55)	√	√		√		√		√	○	RA
Zhou (2022) [104]	China	Longitudinal Study	3583 (aged ≥ 60 and above)	√	√		√		√	√		○	RA

Note: RA: Research article; LR: Literature review; MA: Meta-analysis; OI: Original investigation. SR: Systematic Review; √ denotes the relevant variables appearing in the paper; ○ Indicates that the relevant variables appearing in the paper are associated with the subjective well-being of older people.

## Data Availability

Not applicable.

## Supplementary Materials

The following supporting information can be downloaded at: https://www.mdpi.com/article/10.3390/ijerph192114227/s1, File S1: Study characteristics for the first research theme; File S2: Study characteristics for the second research theme.

## References

[1] Aerts R., Honnay O., Van Nieuwenhuyse A. (2018). Biodiversity and human health: Mechanisms and evidence of the positive health effects of diversity in nature and green spaces. Br. Med. Bull..

[2] Gianfredi V., Buffoli M., Rebecchi A., Croci R., Oradini-Alacreu A., Stirparo G., Marino A., Odone A., Capolongo S., Signorelli C. (2021). Association between Urban Greenspace and Health: A Systematic Review of Literature. Int. J. Environ. Res. Public Health.

[3] The WHOQOL Group (1995). The World Health Organization Quality of Life Assessment (WHOQOL): Position paper from the World Health Organization. Soc. Sci. Med..

[4] Andrews G., Clark M., Luszcz M. (2002). Successful Aging in the Australian Longitudinal Study of Aging: Applying the MacArthur Model Cross-Nationally. J. Soc. Issues.

[5] Reyes-Riveros R., Altamirano A., De La Barrera F., Rozas-Vásquez D., Vieli L., Meli P. (2021). Linking public urban green spaces and human well-being: A systematic review. Urban For. Urban Green..

[6] Sharifi F., Levin I.M., Stone W., Nygaard A. (2021). Green space and subjective well-being in the Just City: A scoping review. Environ. Sci. Policy.

[7] Diener E. (2012). New findings and future directions for subjective well-being research. Am. Psychol..

[8] Phillips A., Khan A., Canters F. (2021). Use-Related and Socio-Demographic Variations in Urban Green Space Preferences. Sustainability.

[9] Chida Y., Steptoe A. (2008). Positive Psychological Well-Being and Mortality: A Quantitative Review of Prospective Observational Studies. Psychosom. Med..

[10] Danner D.D., Snowdon D.A., Friesen W.V. (2001). Positive emotions in early life and longevity: Findings from the nun study. J. Pers. Soc. Psychol..

[11] Alves S., Aspinall P.A., Thompson C.W., Sugiyama T., Brice R., Vickers A. (2008). Preferences of older people for environmental attributes of local parks: The Use of Choice-based Conjoint Analysis. Facilities.

[12] Fry P.S., Debats D.L. (2002). Self-efficacy beliefs as predictors of loneliness and psychological distress in older adults. Int. J. Aging Hum. Dev..

[13] Lyubomirsky S. (2019). The Road to Happiness Is Paved With a Little Gold, a Lot of Reporters, Many E-Mails, Thousands of Frequent Flyer Miles, and 604 Hours of Writing. Perspect. Psychol. Sci..

[14] Müller-Riemenschneider F., Petrunoff N., Sia A., Ramiah A., Ng A., Han J., Wong M., Choo T.B., Uijtdewilligen L. (2018). Prescribing Physical Activity in Parks to Improve Health and Wellbeing: Protocol of the Park Prescription Randomized Controlled Trial. Int. J. Environ. Res. Public Health.

[15] Lee A., Jordan H., Horsley J. (2015). Value of urban green spaces in promoting healthy living and wellbeing: Prospects for planning. Risk Manag. Health Policy.

[16] Gobster P., Westphal L.M. (2004). The human dimensions of urban greenways: Planning for recreation and related experiences. Landsc. Urban Plan..

[17] Markevych I., Schoierer J., Hartig T., Chudnovsky A., Hystad P., Dzhambov A.M., de Vries S., Triguero-Mas M., Brauer M., Nieuwenhuijsen M.J. (2017). Exploring pathways linking greenspace to health: Theoretical and methodological guidance. Environ. Res..

[18] Marselle M.R., Stadler J., Korn H., Irvine K.N., Bonn A. (2019). Biodiversity and Health in the Face of Climate Change.

[19] Lee A.C.K., Maheswaran R. (2010). The health benefits of urban green spaces: A review of the evidence. J. Public Health.

[20] Schipperijn J., Ekholm O., Stigsdotter U.K., Toftager M., Bentsen P., Kamper-Jørgensen F., Randrup T.B. (2010). Factors influencing the use of green space: Results from a Danish national representative survey. Landsc. Urban Plan..

[21] Voigt A., Kabisch N., Wurster D., Haase D., Breuste J. (2014). Structural Diversity: A Multi-dimensional Approach to Assess Recreational Services in Urban Parks. Ambio.

[22] Fuller R.A., Irvine K.N., Devine-Wright P., Warren P.H., Gaston K.J. (2007). Psychological benefits of greenspace increase with biodiversity. Biol. Lett..

[23] Zabelskyte G., Kabisch N., Stasiskiene Z. (2022). Patterns of Urban Green Space Use Applying Social Media Data: A Systematic Literature Review. Land.

[24] Gascon M., Sánchez-Benavides G., Dadvand P., Martínez D., Gramunt N., Gotsens X., Cirach M., Vert C., Molinuevo J.L., Crous-Bou M. (2018). Long-term exposure to residential green and blue spaces and anxiety and depression in adults: A cross-sectional study. Environ. Res..

[25] Egoz S., De Nardi A. (2017). Defining landscape justice: The role of landscape in supporting wellbeing of migrants, a literature review. Landsc. Res..

[26] Mitchell R., Popham F. (2007). Greenspace, urbanity and health: Relationships in England. J. Epidemiol. Community Health.

[27] Maas M. (2020). A Taxonomy of ML for Systems Problems. IEEE Micro.

[28] Mytton O.T., Townsend N., Rutter H., Foster C. (2012). Green space and physical activity: An observational study using Health Survey for England data. Health Place.

[29] De Kleyn L., Mumaw L., Corney H. (2020). From green spaces to vital places: Connection and expression in urban greening. Aust. Geogr..

[30] Song H., Shim C. (2021). Comparing resident and tourist perceptions of an urban park: A latent profile analysis of perceived place value. J. Sustain. Tour..

[31] Van den Berg M., Wendel-Vos W., van Poppel M., Kemper H., van Mechelen W., Maas J. (2015). Health benefits of green spaces in the living environment: A systematic review of epidemiological studies. Urban For. Urban Green..

[32] Battisti L., Pille L., Wachtel T., Larcher F., Säumel I. (2019). Residential Greenery: State of the Art and Health-Related Ecosystem Services and Disservices in the City of Berlin. Sustainability..

[33] Venter Z.S., Barton D.N., Gundersen V., Figari H., Nowell M.S. (2021). Back to nature: Norwegians sustain increased recreational use of urban green space months after the COVID-19 outbreak. Landsc. Urban Plan..

[34] Hami A., Emami F. Spatial Quality of Natural Elements and Safety Perception in Urban Parks. Proceedings of the International Conference on Agricultural, Ecological and Medical Sciences (AEMS-2015).

[35] Shuster J.J. (2011). Review: Cochrane handbook for systematic reviews for interventions, Version 5.1.0, published 3/2011. Julian, P.T. Higgins and Sally Green, Editors. Res. Synth. Methods..

[36] Van Eck N.J., Waltman L. (2017). Citation-based clustering of publications using CitNetExplorer and VOSviewer. Scientometrics.

[37] Orduña-Malea E., Costas R. (2021). Link-based approach to study scientific software usage: The case of VOSviewer. Scientometrics.

[38] Huang Y.-J., Cheng S., Yang F.-Q., Chen C. (2022). Analysis and Visualization of Research on Resilient Cities and Communities Based on VOSviewer. Int. J. Environ. Res. Public Health.

[39] Oyewola D.O., Dada E.G. (2022). Exploring machine learning: A scientometrics approach using bibliometrix and VOSviewer. SN Appl. Sci..

[40] Meng L., Wen K.-H., Brewin R., Wu Q. (2020). Knowledge Atlas on the Relationship between Urban Street Space and Residents’ Health—A Bibliometric Analysis Based on VOSviewer and CiteSpace. Sustainability.

[41] Kabisch N., Frantzeskaki N., Pauleit S., Naumann S., Davis M., Artmann M., Bonn A. (2016). Nature-based solutions to climate change mitigation and adaptation in urban areas: Perspectives on indicators, knowledge gaps, barriers, and opportunities for action. Ecol. Soc..

[42] van den Bogerd N., Dijkstra S.C., Koole S.L., Seidell J.C., de Vries R., Maas J. (2020). Nature in the indoor and outdoor study environment and secondary and tertiary education students’ well-being, academic outcomes, and possible mediating pathways: A systematic review with recommendations for science and practice. Health Place.

[43] Silva R.A., Rogers K., Buckley T.J. (2018). Advancing environmental epidemiology to assess the beneficial influence of the natural environment on human health and well-being. Environ. Sci. Technol..

[44] Wang R., Helbich M., Yao Y., Zhang J., Liu P., Yuan Y., Liu Y. (2019). Urban greenery and mental wellbeing in adults: Cross-sectional mediation analyses on multiple pathways across different greenery measures. Environ. Res..

[45] Xu Z., Zhang W., Zhang X., Wang Y., Chen Q., Gao B., Li N. (2022). Multi-Level Social Capital and Subjective Wellbeing Among the Elderly: Understanding the Effect of Family, Workplace, Community, and Society Social Capital. Front. Public Health.

[46] Jennings V., Bamkole O. (2019). The relationship between social cohesion and urban green space: An avenue for health promotion. Int. J. Environ. Res. Public Health.

[47] Ayala-Azcárraga C., Diaz D., Zambrano L. (2019). Characteristics of urban parks and their relation to user well-being. Landsc. Urban Plan..

[48] Miralles-Guasch C., Dopico J., Delclòs-Alió X., Knobel P., Marquet O., Maneja-Zaragoza R., Schipperijn J., Vich G. (2019). Natural Landscape, Infrastructure, and Health: The Physical Activity Implications of Urban Green Space Composition among the Elderly. Int. J. Environ. Res. Public Health.

[49] Palliwoda J., Priess J.A. (2021). What do people value in urban green? Linking characteristics of urban green spaces to users’ perceptions of nature benefits, disturbances, and disservices. Ecol. Soc..

[50] Veitch J., Ball K., Rivera E., Loh V., Deforche B., Best K., Timperio A. (2022). What entices older adults to parks? Identification of park features that encourage park visitation, physical activity, and social interaction. Landsc. Urban Plan..

[51] Ettema D., Schekkerman M. (2016). How do spatial characteristics influence well-being and mental health? Comparing the effect of objective and subjective characteristics at different spatial scales. Travel Behav. Soc..

[52] Levy-Storms L., Chen L., Loukaitou-Sideris A. (2017). Older Adults’ Needs and Preferences for Open Space and Physical Activity in and Near Parks: A Systematic Review. J. Aging Phys. Act..

[53] Kabisch N., Qureshi S., Haase D. (2015). Human–environment interactions in urban green spaces—A systematic review of contemporary issues and prospects for future research. Environ. Impact Assess. Rev..

[54] Wen C., Albert C., Von Haaren C. (2018). The elderly in green spaces: Exploring requirements and preferences concerning nature-based recreation. Sustain. Cities Soc..

[55] Ali J., Rahaman M., Hossain S.I. (2022). Urban green spaces for elderly human health: A planning model for healthy city living. Land Use Policy.

[56] Gutiérrez M.F., Noguez A.B., Flamenco-Sandoval A., Serrano A.M., Flores-Torres A., Ramírez A.K.G., Alcántara C. (2021). Availability and accessibility of urban green spaces: The case of the urban zone of Queretaro Metropolitan Area, Mexico. J. Maps..

[57] Macintyre V.G., Cotterill S., Anderson J., Phillipson C., Benton J.S., French D.P. (2019). “I Would Never Come Here Because I’ve Got My Own Garden”: Older Adults’ Perceptions of Small Urban Green Spaces. Int. J. Environ. Res. Public Health.

[58] Bertram C., Rehdanz K. (2015). The role of urban green space for human well-being. Ecol. Econ..

[59] Akpinar A. (2016). How is quality of urban green spaces associated with physical activity and health?. Urban For. Urban Green..

[60] Rioux L., Werner C.M., Mokounkolo R., Brown B.B. (2016). Walking in two French neighborhoods: A study of how park numbers and locations relate to everyday walking. J. Environ. Psychol..

[61] Larson L., Jennings V., Cloutier S.A. (2016). Public Parks and Wellbeing in Urban Areas of the United States. PLoS ONE.

[62] Tilley S., Neale C., Patuano A., Cinderby S. (2017). Older People’s Experiences of Mobility and Mood in an Urban Environment: A Mixed Methods Approach Using Electroencephalography (EEG) and Interviews. Int. J. Environ. Res. Public Health.

[63] Zhang S., Lin S., Li Z., Guo Y. (2019). Influence of Neighborhood Environmental Perception on Self-Rated Health of Residents in Cities of China a Case Study of Wuhan. Hum. Geogr..

[64] Nishigaki M., Hanazato M., Koga C., Kondo K. (2020). What Types of Greenspaces Are Associated with Depression in Urban and Rural Older Adults? A Multilevel Cross-Sectional Study from JAGES. Int. J. Environ. Res. Public Health.

[65] Dennis M., Cook P.A., James P., Wheater C.P., Lindley S.J. (2020). Relationships between health outcomes in older populations and urban green infrastructure size, quality and proximity. BMC Public Health.

[66] Shuvo F.K., Feng X., Astell-Burt T. (2020). Urban green space quality and older adult recreation: An international comparison. Cities Health.

[67] Nguyen P.-Y., Astell-Burt T., Rahimi-Ardabili H., Feng X. (2021). Green Space Quality and Health: A Systematic Review. Int. J. Environ. Res. Public Health.

[68] Roberts M., Irvine K.N., McVittie A. (2021). Associations between greenspace and mental health prescription rates in urban areas. Urban For. Urban Green..

[69] Petrunoff N.A., Yi N.X., Dickens B., Sia A., Koo J., Cook A.R., Lin W.H., Ying L., Hsing A.W., van Dam R.M. (2021). Associations of park access, park use and physical activity in parks with wellbeing in an Asian urban environment: A cross-sectional study. Int. J. Behav. Nutr. Phys. Act..

[70] Oviedo M., Drescher M., Dean J. (2022). Urban greenspace access, uses, and values: A case study of user perceptions in metropolitan ravine parks. Urban For. Urban Green..

[71] White M.P., Pahl S., Wheeler B.W., Depledge M.H., Fleming L.E. (2017). Natural environments and subjective wellbeing: Different types of exposure are associated with different aspects of wellbeing. Health Place.

[72] Cao X. (2016). How does neighborhood design affect life satisfaction? Evidence from Twin Cities. Travel Behav. Soc..

[73] Reid C.E., Clougherty J.E., Shmool J.L., Kubzansky L.D. (2017). Is All Urban Green Space the Same? A Comparison of the Health Benefits of Trees and Grass in New York City. Int. J. Environ. Res. Public Health.

[74] Duan J., Wang Y., Chen F., Xia B. (2018). Perception of Urban Environmental Risks and the Effects of Urban Green Infrastruc-tures (UGIs) on Human Well-Being in Four Public Green Spaces of Guangzhou, China. Environ. Manag..

[75] Benton J.S., Anderson J., Cotterill S., Dennis M., Lindley S.J., French D.P. (2018). Evaluating the impact of improvements in urban green space on older adults’ physical activity and wellbeing: Protocol for a natural experimental study. BMC Public Health.

[76] Cameron R.W.F., Brindley P., Mears M., McEwan K., Ferguson F., Sheffield D., Jorgensen A., Riley J., Goodrick J., Ballard L. (2020). Where the wild things are! Do urban green spaces with greater avian biodiversity promote more positive emotions in humans?. Urban Ecosyst..

[77] Sundevall E., Jansson M. (2020). Inclusive Parks across Ages: Multifunction and Urban Open Space Management for Children, Adolescents, and the Elderly. Int. J. Environ. Res. Public Health.

[78] Houlden V., de Albuquerque J.P., Weich S., Jarvis S. (2021). Does nature make us happier? A spatial error model of greenspace types and mental wellbeing. Environ. Plan. B Urban Anal. City Sci..

[79] Jabbar M., Yusoff M.M., Shafie A. (2021). Assessing the role of urban green spaces for human well-being: A systematic review. GeoJournal.

[80] Ha J., Kim H.J., With K.A. (2021). Urban green space alone is not enough: A landscape analysis linking the spatial distribution of urban green space to mental health in the city of Chicago. Landsc. Urban Plan..

[81] Cottagiri S.A., Villeneuve P.J., Raina P., Griffith L.E., Rainham D., Dales R., Peters C.E., Ross N.A., Crouse D.L. (2022). Increased urban greenness associated with improved mental health among middle-aged and older adults of the Canadian Longitudinal Study on Aging (CLSA). Environ. Res..

[82] Wen C., Albert C., von Haaren C. (2022). Nature-based recreation for the elderly in urban areas: Assessing opportunities and demand as planning support. Ecol. Process..

[83] Kühn S., Düzel S., Mascherek A., Eibich P., Krekel C., Kolbe J., Goebel J., Gallinat J., Wagner G.G., Lindenberger U. (2021). Urban green is more than the absence of city: Structural and functional neural basis of urbanicity and green space in the neighbourhood of older adults. Landsc. Urban Plan..

[84] Zhai Y., Baran P.K. (2017). Urban park pathway design characteristics and senior walking behavior. Urban For. Urban Green..

[85] Cinderby S., Cambridge H., Attuyer K., Bevan M., Croucher K., Gilroy R., Swallow D. (2018). Co-designing Urban Living Solutions to Improve Older People’s Mobility and Well-Being. J. Urban Health.

[86] Ottoni C.A., Sims-Gould J., Winters M. (2021). Safety perceptions of older adults on an urban greenway: Interplay of the social and built environment. Health Place.

[87] Kou R., Hunter R.F., Cleland C., Ellis G. (2021). Physical environmental factors influencing older adults’ park use: A qualitative study. Urban For. Urban Green..

[88] Kimic K., Polko P. (2022). The Use of Urban Parks by Older Adults in the Context of Perceived Security. Int. J. Environ. Res. Public Health.

[89] Rahm J., Sternudd C., Johansson M. (2021). “In the evening, I don’t walk in the park”: The interplay between street lighting and greenery in perceived safety. Urban Des. Int..

[90] Cerin E., Nathan A., van Cauwenberg J., Barnett D.W., Barnett A. (2017). The neighbourhood physical environment and active travel in older adults: A systematic review and meta-analysis. Int. J. Behav. Nutr. Phys. Act..

[91] Wang F., Wang D. (2015). Research Progress on Subjective Well-Being Metrics and Its Implications for Implications for Smart City Construction. Prog. Geogr..

[92] Forbes M.K., Spence K.M., Wuthrich V.M., Rapee R.M. (2015). Mental Health, and Well-being of Older Workers in Australia. Work Aging Retire..

[93] Sang Å.O., Knez I., Gunnarsson B., Hedblom M. (2016). The effects of naturalness, gender, and age on how urban green space is perceived and used. Urban For. Urban Green..

[94] Gobbens R.J.J., Assen M.A.L.M.V. (2018). Associations of Environmental Factors With Quality of Life in Older Adults. Gerontologist.

[95] Janus E., Smrokowska-Reichmann A. (2019). Level of happiness and happiness-determining factors perceived by women aged over 60 years. J. Women Aging..

[96] Batz C., Tay L., Diener E., Oishi S., Tay L. (2018). Gender Differences in Subjective Well-Being. Handbook of Well-Being.

[97] Massey B., Edwards A.V., Musikanski L. (2021). Life Satisfaction, Affect, and Belonging in Older Adults. Appl. Res. Qual. Life.

[98] Astell-Burt T., Feng X. (2019). Association of Urban Green Space With Mental Health and General Health Among Adults in Australia. JAMA Netw. Open..

[99] Li X., Liu L., Zhang Z., Zhang W., Liu D., Feng Y. (2020). Gender Disparity in Perceived Urban Green Space and Subjective Health and Well-Being in China: Implications for Sustainable Urban Greening. Sustainability.

[100] Enssle F., Kabisch N. (2020). Urban green spaces for the social interaction, health and well-being of older people— An integrated view of urban ecosystem services and socio-environmental justice. Environ. Sci. Policy.

[101] Xie L., Yao Y.-D., Tang L.-L., Zhang S., Yang H.-L., Zhang S.-Q., Wu Y.-Y., Li Z.-Y. (2021). Effect of Working After Retirement on the Mental Health of Older People: Evidence from China. Front. Psychiatry.

[102] Hackert M.Q.N., van Exel J., Brouwer W.B.F. (2021). Content validation of the Well-being of Older People measure (WOOP). Health Qual. Life Outcomes.

[103] Han B., Li D., Chang P.-J. (2021). The effect of place attachment and greenway attributes on well-being among older adults in Taiwan. Urban For. Urban Green..

[104] Zhou R., Zheng Y.-J., Yun J.-Y., Wang H.-M. (2022). The Effects of Urban Green Space on Depressive Symptoms of Mid-Aged and Elderly Urban Residents in China: Evidence from the China Health and Retirement Longitudinal Study. Int. J. Environ. Res. Public Health.

[105] Polko P., Kimic K. (2022). Gender as a factor differentiating the perceptions of safety in urban parks. Ain. Shams Eng. J..

[106] Huerta C.M., Cafagna G. (2021). Snapshot of the Use of Urban Green Spaces in Mexico City during the COVID-19 Pandemic: A Qualitative Study. Int. J. Environ. Res. Public Health.

[107] Van Houwelingen-Snippe J., Van Rompay T.J.L., Ben Allouch S. (2020). Feeling Connected after Experiencing Digital Nature: A Survey Study. Int. J. Environ. Res. Public Health.

[108] Van Herzele A., de Vries S. (2012). Linking green space to health: A comparative study of two urban neighbourhoods in Ghent, Belgium. Popul. Environ..

[109] Morgan M., Fenner R. (2019). Spatial evaluation of the multiple benefits of sustainable drainage systems. Water Manag..

[110] Fei W., Opoku A., Agyekum K., Oppon J.A., Ahmed V., Chen C., Lok K.L. (2021). The Critical Role of the Construction Industry in Achieving the Sustainable Development Goals (SDGs): Delivering Projects for the Common Good. Sustainability.

[111] Lee K., Dabelko-Schoeny H., Jedlicka H., Burns T. (2019). Older Adults’ Perceived Benefits of Equine-Assisted Psychotherapy: Implications for Social Work. Res. Soc. Work Pract..

[112] Cramm J.M., van Dijk H.M., Nieboer A.P. (2013). The Importance of Neighborhood Social Cohesion and Social Capital for the Well Being of Older Adults in the Community. Gerontol..

[113] Ding Y., Shi B., Su G., Li Q., Meng J., Jiang Y., Qin Y., Dai L., Song S. (2021). Assessing Suitability of Human Settlements in High-Altitude Area Using a Comprehensive Index Method: A Case Study of Tibet, China. Sustainability.

[114] Burwell-Naney K., Wilson S.M., Whitlock S.T., Puett R. (2019). Hybrid Resiliency-Stressor Conceptual Framework for Informing Decision Support Tools and Addressing Environmental Injustice and Health Inequities. Int. J. Environ. Res. Public Health.

[115] Lee Y., Chon D., Kim J., Ki S., Yun J. (2020). The Predictive Value of Social Frailty on Adverse Outcomes in Older Adults Living in the Community. J. Am. Med. Dir. Assoc..

[116] Jim C. (2004). Green-space preservation and allocation for sustainable greening of compact cities. Cities.

[117] Kubiszewski I., Zakariyya N., Costanza R. (2018). Objective and Subjective Indicators of Life Satisfaction in Australia: How Well Do People Perceive What Supports a Good Life?. Ecol. Econ..

[118] Matsuoka R.H., Kaplan R. (2008). People needs in the urban landscape: Analysis of Landscape And Urban Planning contributions. Landsc. Urban Plan..

[119] Barbosa O., Tratalos J.A., Armsworth P.R., Davies R.G., Fuller R.A., Johnson P., Gaston K.J. (2007). Who benefits from access to green space? A case study from Sheffield, UK. Landsc. Urban Plan..

[120] Calogiuri G., Chroni S. (2014). The impact of the natural environment on the promotion of active living: An integrative systematic review. BMC Public Health.

[121] Pedell S., Borda A., Keirnan A., Aimers N. (2021). Combining the Digital, Social and Physical Layer to Create Age-Friendly Cities and Communities. Int. J. Environ. Res. Public Health.

[122] Kimic K., Ostrysz K. (2021). Assessment of Blue and Green Infrastructure Solutions in Shaping Urban Public Spaces—Spatial and Functional, Environmental, and Social Aspects. Sustainability.

[123] A Greenfield E., Black K., Buffel T., Yeh J. (2018). Community Gerontology: A Framework for Research, Policy, and Practice on Communities and Aging. Gerontologist.

[124] Perry M., Cotes L., Horton B., Kunac R., Snell I., Taylor B., Wright A., Devan H. (2021). “Enticing” but Not Necessarily a “Space Designed for Me”: Experiences of Urban Park Use by Older Adults with Disability. Int. J. Environ. Res. Public Health.

[125] Reid C.E., Rieves E.S., Carlson K. (2022). Perceptions of green space usage, abundance, and quality of green space were associated with better mental health during the COVID-19 pandemic among residents of Denver. PLoS ONE.

[126] Appau S., Churchill S.A., Farrell L. (2019). Social integration and subjective wellbeing. Appl. Econ..

[127] Aravena J.K.S., Blanco R.M.N., Coria M.D.C.D., Lagos L.A. (2020). Significado de bienestar subjetivo e inclusión económica en adultos mayores líderes de asociaciones en el sur de Chile. Interdiscip. Rev. Psicol. Cienc. Afines.

[128] van Kamp I., van Loon J., Droomers M., de Hollander A. (2004). Residential Environment and Health: A Review of Methodological and Conceptual Issues. Rev. Environ. Health.

[129] Dadvand P., Hariri S., Abbasi B., Heshmat R., Qorbani M., Motlagh M.E., Basagaña X., Kelishadi R. (2019). Use of green spaces, self-satisfaction and social contacts in adolescents: A population-based CASPIAN-V study. Environ. Res..

[130] Espinoza J.B.R., Hernández M.D.L.G., Becerril L.C., Galindo L.V., Kempfer S.S. (2018). ADAPTACIÓN DEL MODELO DE KRISTEN SWANSON PARA EL CUIDADO DE ENFERMERÍA EN ADULTAS MAYORES. Texto Context. Enferm..

[131] Mackett R. (2014). Has the policy of concessionary bus travel for older people in Britain been successful?. Case Stud. Transp. Policy.

[132] Cohen B., Lawrence K.T., Armstrong A., Wilcha M., Gatti A. (2018). Greening Lafayette: A model for building sustainable community. Int. J. Sustain. High. Educ..

[133] Kandt J., Leak A. (2019). Examining inclusive mobility through smartcard data: What shall we make of senior citizens’ declining bus patronage in the West Midlands?. J. Transp. Geogr..

[134] Gao T., Zhang T., Zhu L., Qiu L. (2019). Exploring Psychophysiological Restoration and Individual Preference in the Different Environments Based on Virtual Reality. Int. J. Environ. Res. Public Health.

[135] Lak A., Rashidghalam P. (2021). Improving the Elderly’s Mental Health by Using Public Open Spaces in Disadvantaged Urban Neighbor-hoods: Tehran, Iran.

