# Supplementary material for: Urban Green Space and Subjective Well-Being of Older People: A Systematic Literature Review
Source: Int J Environ Res Public Health. 2022 Oct 31;19(21):14227. doi: 10.3390/ijerph192114227 (PMC9653953; doi:10.3390/ijerph192114227)
Supplement: Supplementary file 1 [file ijerph-19-14227-s001.zip › ijerph-1893778-supplementary.pdf]

Supplementary File S1: Study characteristics for the first research theme.

First research theme (About UGS characteristics and SWB)			Articles	
			Number	Percentage
Region	Europe		24	48%
	Asia		11	22%
	America		10	20%
	Oceania		5	10%
		Total	50	100%
Research content	Spatial characteristics		19	38%
	Green characteristics		15	30%
	Grey characteristics		9	18%
	Spatial characteristics、Green characteristics、Grey characteristics		4	8%
	Spatial characteristics、Green characteristics		1	2%
	Green characteristics、Grey characteristics		1	2%
	Spatial characteristics、Grey characteristics		1	2%
		Total	50	100%
Research method	Quantitative	survey	12	68%
		questionnaire	10	
		Regression analysis	7	
		Cross-sectional	5	
	Qualitative	bibliometric method	7	30%
		Case study	2	
		Interviews	5	
		Meta analysis	1	
	Mixed method	Social prescribing approach	1	2%
		Total	50	100%

Supplementary File S2: Study characteristics for the second research theme.

Second research theme (About UGS characteristics and SWB)			Articles	
			Number	Percentage
Region	Europe		5	48%
	Asia		3	22%
	America		3	20%
	Oceania		4	10%
		Total	15	100%
Research method	Quantitative	Survey	5	80%
		Questionnaire	3	
		Cohort study	1	
		Cross-sectional	1	
		Longitudinal Study	1	
		Ordinal regression	1	
	Qualitative	Review	1	20%
		Case study	1	
		Bibliometric approach	1	
		Total	15	100%
